# SOCIOEMOTIONAL EXPERIENCE IN CLOSE PERSONAL RELATIONSHIPS AND TRAJECTORIES OF COGNITIVE AGING

**DOI:** 10.1093/geroni/igac059.463

**Published:** 2022-12-20

**Authors:** Sara Moorman, Lucas Hamilton

## Abstract

Linked lives is a key tenet of the life course framework: Individuals age and develop in the company of a social convoy, or core set of relationships. The quality and quantity of relationships with friends and family are well-known predictors of physical and mental health outcomes, with research on how relationships affect cognitive health just beginning to blossom. This symposium presents four sociological studies of how positive and negative experiences in central, long-term personal relationships – marriages, parent-adult child relationships, and friendships – relate to cognition and the development of cognitive impairment over long periods of the life course, using data from the Health and Retirement Study (HRS) and the Wisconsin Longitudinal Study (WLS). Stokes, Prasad, and Barooah find that experiences of loneliness in marriage are negatively related both to one’s own cognition and to the spouse’s cognition. Herd and Sicinski also highlight potential negative and gendered aspects of marriage, showing no differences in cognitive performance between married and single men, while married women’s cognition is not as strong as single women’s cognition. In parent-adult child relationships, Zhang and Liu demonstrate that social support has stronger positive effects, and social strain, stronger negative effects, for mothers as compared to fathers. Moorman and Pai examine friendships with non-kin, and find benefits of emotional and instrumental support to cognition in the long term. Discussant Lucas Hamilton will provide perspective from psychology, addressing ambivalence in relationships and the potential for bidirectional associations between social experience and cognitive function.

